# Factors Affecting the Mortality Rate in Non-COVID-19 Intensive Care Unit Patients During the COVID-19 Pandemic in Cyprus: A Retrospective Cohort Study

**DOI:** 10.7759/cureus.47610

**Published:** 2023-10-24

**Authors:** Omer Tasargol

**Affiliations:** 1 Anesthesiology, Dr. Burhan Nalbantoglu State Hospital, Nicosia, CYP

**Keywords:** pandemic, covid-19, time of admission, mortality rate, intensive care unit

## Abstract

Introduction: Mortality statistics constitute a pivotal element in informing public health policymaking in critical care settings. Mortality rates exhibit temporal variability, and their quantification is susceptible to well-established biases that have been exacerbated in the backdrop of the COVID-19 pandemic. A multitude of factors contribute to the process of patients’ outcomes within the intensive care unit (ICU) setting. The primary aim of this study is to compare the mortality rate observed during the initial and subsequent phases of the COVID-19 pandemic in non-COVID-19 patient cohorts. Secondary objectives encompass evaluating the demographic and clinical factors and admission times to the ICU as an independent predictor affecting mortality.

Methods and materials: A retrospective investigation of the data gathered from 1127 non-COVID-19 patients admitted to an ICU situated in Nicosia, Cyprus between March 2020 and December 2022 was performed. We divided the study period into two distinct timeframes. The first period spanned from the onset of the COVID-19 pandemic up until January 2021, coinciding with the relaxation of COVID-related restrictions. The second period was defined as the period when restrictions were not applied. The time of admission to the ICU is categorized as either off-hours or business hours. We recorded various patient characteristics, including age, gender, Acute Physiology and Chronic Health Evaluation II (APACHE II), Glasgow Coma Scale (GCS), Sequential Organ Failure Assessment (SOFA) scores, hospitalization duration, discharge details, mortality events with precise timestamps and primary diagnosis for admission. Multivariate logistic regression analysis was performed with these characteristics to predict the likelihood of mortality.

Results: This study included 632 males (56.1%) and 495 females (43.9%). Within the patient cohort, 653 patients (57.9%) were discharged from the ICU, while 474 patients (42.1%) experienced mortality during their ICU stay. No significant correlation was found whether patients were admitted to ICU during the first or second period of the COVID-19 pandemic. There was a significant difference in the comparison of outcomes within the ICU between the off-hours and business hours (p=0.001). A total of 329 of 618 (53.2%) patients admitted in off-hours and 145 of 509 (28.4%) patients admitted in business hours died. Moreover, the mean GCS, APACHE II and SOFA scores were higher in patients admitted during off-hours. APACHE II score (OR: 1.11, 95% CI: 1.06 to 1.15, p<0.01), SOFA (OR: 1.21, 95% CI: 1.10 to 1.31, p<0.01) and GCS (OR: 0.88, 95% CI: 0.84 to 0.92, p<0.01) scores and admission to the ICU in off-hours 2.63 (1.91-3.67) were significantly associated with mortality.

Conclusion: The results of this retrospective cohort analysis have shown that the mortality rate was higher in non-COVID-19 patients admitted to ICU during off-hours compared to those admitted during business hours. However, no significant difference was found in the mortality rate between the admissions during the first and second periods of the COVID-19 pandemic.

## Introduction

Critical care is a specialized domain within the field of medicine which pertains to the comprehensive evaluation, treatment, and subsequent continuous monitoring of critically ill patients. Recently, there has been notable progress observed in the realm of critical care, characterized by significant strides in medical knowledge, technological advancements, and the development of clinical expertise [1]. An intensive care unit (ICU) represents an exceptionally specialized and meticulously organized critical healthcare facility [2]. It is purposefully configured, staffed with specialized personnel, strategically located, furnished, equipped, and exclusively dedicated to providing care and managing individuals afflicted by severe injuries or intricate medical conditions that necessitate intensive medical interventions and continuous monitoring of vital signs and responses to treatment around the clock for 24 hours [3].

As the global COVID-19 pandemic continues to unfold, the critical care community faces the imperative task of prepping for the intricate challenges it brings [4]. The COVID-19 crisis underscores the pivotal role played by ICUs, but it also highlights certain constraints, including spatial limitations and staffing shortages. Establishing a globally oriented framework for the future critical care system is of paramount importance. In light of the worldwide proliferation of the COVID-19 pandemic, ICU practitioners, hospital administrators, governmental bodies, policymakers, and researchers must prepare for an upsurge in critically ill patients [5,6]. The collective experience of ICUs managing COVID-19 outbreaks offers valuable insights.

Numerous determinants influence admission and discharge decisions within the purview of ICUs. These considerations encompass aspects such as the repercussions of the underlying medical condition, the availability of specialized therapeutic resources, the anticipated impact of treatment on the disease's progression, the pros and cons of therapeutic interventions, and the overarching strategic approach of the ICU itself [7-9]. Moreover, studies have reported that staffing levels, among many other factors in the hospital setting, contribute to adverse patient outcomes [10-12]. Staffing levels often dwindle during non-standard shifts, including evenings, nights, weekends, and holidays, collectively referred to as 'off-hours,' despite the continuous, round-the-clock intensive care typically demanded by ICU patients. Furthermore, these time periods may witness restricted access to various medical equipment, potentially resulting in extended durations for specific diagnostic and therapeutic procedures, or in some cases, necessitating deferral of such interventions until standard working hours are reinstated.

Prior investigations involving hospitalized critically ill patients have indicated that disparities in mortality rates between admissions occurring on weekdays and those on weekends are most pronounced among individuals afflicted with intricate medical conditions carrying elevated mortality risks [10,11]. Moreover, several studies focus on the specified mortality rates within certain time frames. For this purpose, the first 24 hours, 48 hours, and 72 hours, as well as weekly and 28-day mortality rates, are among the most used frames [13-15]. Additionally, previous studies performed before the COVID-19 pandemic reported an association between ICU admissions during 'off-hours' and heightened mortality rates [10-12]. However, no previous studies have examined this aspect in the context of the COVID-19 pandemic, specifically whether the risk of ICU mortality remains consistent between daytime and nighttime admissions as well as between weekends and weekdays. COVID-19-positive patients were excluded from this study, recognizing that the contagiousness and lethality of variants within COVID-19 ICU may introduce significant variations. Instead, we focused on non-COVID-19 patients due to potential delays resulting from the stringent measures implemented in COVID-19 ICU, complications arising from unconventional treatment approaches such as frequent repositioning and infrequently used medications, as well as diagnostic delays and complications attributable to the constantly evolving spectrum of COVID-19 related symptoms.

With the COVID-19 pandemic, there have been serious changes in ICU practice, as in every field of medicine. Moreover, the COVID-19 pandemic has led to an unprecedented impact on healthcare services. It is reasonable to presume that the healthcare staff's physical and psychological well-being may have influenced ICU outcomes during the first period of restrictions. Considering these factors, the COVID-19 pandemic was divided into two periods. The first period of the pandemic represents which lockdowns and restrictions were strictly applied and only emergency patients were admitted to our hospital, between the onset of the COVID-19 pandemic and January 2021. The second period was defined as the period when restrictions were not applied. There is a paucity of data available for comparing the mortality rates in non-COVID ICUs between the first and second periods of the COVID-19 pandemic.

It is hypothesized that mortality rates might increase among non-COVID-19 patients admitted to the ICU during the COVID-19 pandemic period due to the lower staffing levels and potentially suboptimal care practices. To assess the impact of reduced ICU personnel and related factors on the death rate, we conducted a retrospective cohort study using prospectively collected data from an ICU located in Nicosia, Cyprus. The primary aim of this present study was to compare the mortality rate between the first and second periods of the COVID-19 pandemic in non-COVID patients. Secondary objectives include examining potential disparities in ICU length of stay and the utilization of mechanical ventilation (MV) between patients admitted during business hours and those admitted during off-hours, as well as assessing the impact of demographic and clinical factors on ICU mortality.

## Materials and methods

Ethical board approval of this retrospective study (Reference: YTK.1.01-EK26/23) was obtained from Northern Cyprus Ministry of Health’s Dr. Burhan Nalbantoglu State Hospital Ethic Committee, ensuring that all aspects of the research adhered to the ethical standards. In our hospital setting, physicians, nurses, and ICU staff were working in eight-hour shifts in the COVID-19 ICU which could affect the methodology of the investigation; therefore, COVID-19 patients and ICUs were excluded from the study. This comprehensive data collection approach ensured a robust foundation for our retrospective analysis. The study period spanned from March 2020 to December 31, 2022. To gather data, we meticulously accessed various sources, including the hospital's automation systems and intensive care follow-up forms. This rigorous approach ensured the acquisition of complete and accurate patient information, upholding the integrity and reliability of our study's findings.

Outcomes

We recorded various patient characteristics as independent variables, including age, gender, the Acute Physiology and Chronic Health Evaluation-II (APACHE II) score, the Sequential Organ Failure Assessment (SOFA) score, the Glasgow Coma Scale (GCS), hospitalization duration, discharge details, mortality events with precise timestamps (including days and hours) and primary diagnosis for admission.

APACHE-II score was developed to assess the disease severity of ICU patients and predict their likelihood of mortality. This score is calculated based on parameters like body temperature, heart rate, respiratory rate, mean arterial pressure, and pH, with a range from 0 to 71. A higher score is indicative of an elevated risk of hospital death [16]. The SOFA score, on the other hand, was designed to evaluate organ dysfunction in critically ill patients. It assesses six organ systems: pulmonary, blood, cardiovascular, hepatic, central nervous, and renal. The SOFA score systematically and continuously monitors the condition of these organs during the patient's stay in the ICU. In the SOFA scoring system, each of the six examined organs is assigned a score from 0 to 4 based on the extent of dysfunction. Since its introduction, the SOFA score has been routinely used in ICUs to predict patient morbidity and mortality [17]. The GCS score was computed in a conventional manner, summing scores for eye-opening [1-4], verbal response [1-5], and motor response [1-6], with a possible range from 3 to 15 [18]. The GCS score was assessed upon admission to the ICU, while the APACHE-II and SOFA scores were determined on the first day of treatment. The calculations for APACHE-II and SOFA scores were performed using an online calculator.

Temporal analysis

The study period was divided into two distinct time frames. The first period spanned from the onset of the COVID-19 pandemic up until January 2021, in which lockdowns and restrictions were strictly applied and only emergency patients were admitted to our hospital. As restrictions and lockdown policies began to ease, the period after January 2021 was defined as the second period. This temporal division allowed us to conduct additional analyses, exploring potential temporal trends or shifts in patient characteristics and outcomes during these delineated periods. This approach enabled us to capture potential changes in healthcare practices and patient demographics over time, enhancing the richness of our findings.

Statistical analysis

For the statistical analysis, we utilized the NCSS (Number Cruncher Statistical System) 2007 (NCSS LLC, Kaysville, USA) and PASS (Power Analysis and Sample Size) 2008 (NCSS LLC, Kaysville, USA) statistical software. Descriptive statistics, including means and standard deviations, were used. Student's t-test was used to compare parameters with a normal distribution. For parameters that did not conform to a normal distribution, we employed the Mann-Whitney U test. Categorical parameters were assessed using the Pearson chi-square test. Multivariate logistic regression analysis was performed with characteristics including age, sex, length of MV time and ICU stay, GCS, SOFA and APACHE II scores, the primary reason for admission and admission time to the ICU. An odds ratio (OR) with a 95% confidence interval (CI) was calculated. We considered statistical significance to be present when differences yielded a p-value less than 0.05.

## Results

This study involved a cohort of 1,127 patients, comprising 632 males (56.1%) and 495 females (43.9%). The age of these patients ranged from 18 to 99 years, with a mean age of 58.7±20.2 years. Summary statistics for patient scores on the APACHE II, GCS, SOFA, the average length of MV, ICU stay, and the distribution of death by admission time are presented in Table 1. Within the patient cohort, 653 patients (57.9%) were discharged from the ICU, while 474 patients (42.1%) experienced mortality during their ICU stay.

**Table 1:** Length of ICU stay, length of MV time, and the average scores for ICU. Acute Physiology and Chronic Health Evaluation II (APACHE II); Glasgow Coma Scale (GCS); Sequential Organ Failure Assessment (SOFA); intensive care unit (ICU); mechanic ventilation (MV); standard deviation (SD)

		Min-Max	Mean±SD
Length of ICU stay (days)		1-134	7.03 ±10.0
Age (years)		18-99	58.7 ± 20.2
Length of MV time (days)		1-133	4.03 ± 7.59
APACHE II		4-37	18.70 ± 6.80
SOFA		0-21	6.33 ± 3.45
GCS		3-15	10.21 ± 4.42
		n	%
n=1127	Discharged	653	57.9
	Died	474	42.1
n=1127	Female	465	43.9
	Male	632	56.1

When examining the days of ICU admission, significant differences in mortality rates were observed among admission times. There was a significant difference in the comparison of outcomes within the ICU between periods classified as off-hours and business hours (p=0.001). A total of 329 of 618 (53.2%) patients admitted in off-hours and 145 of 509 (28.4%) patients admitted in business hours died. Moreover, the mean scores of GCS, APACHE II and SOFA were higher in patients admitted during off-hours compared to business hours (Table 2).

**Table 2:** Clinical outcomes of patients by time of admission Acute Physiology and Chronic Health Evaluation II (APACHE II); Glasgow Coma Scale (GCS); Sequential Organ Failure Assessment (SOFA); intensive care unit (ICU); mechanic ventilation (MV); standard deviation (SD) Others refer to post-operative, infectious disease, kidney failure and gastrointestinal disease; Business hours referred to 8:00-16:59 on weekdays; Off-hours referred to 17:00-07:59 on weekdays and weekends (from 00:00 Saturday to 23:59 Sunday).

	Off-hours (n=618)	Business hours (n=509)	p
Age (years)	59.36±20.08	57.83±20.27	0.203
Gender			
Female	269 (43.5%)	226 (44.4%)	0.769
Male	349 (56.5%)	283 (55.6%)	
ICU outcome			
Died	329 (53.2%)	145 (28.4%)	<0.001
Discharged	289 (46.8%)	364 (71.6%)	
Length of ICU stay (days)	7.26±10.88	6.75±8.82	0.393
Length of MV time (days)	4.49±7.96	3.47±7.09	0.025
APACHE II score	19.66±6.98	17.61±6.40	<0.001
GCS score	9.67±4.56	10.83±4.28	<0.001
SOFA score	6.83±3.59	5.72±3.18	<0.001
Primary reason for admission			0.169
Cerebrovascular disease	100 (16.1%)	94 (18.4%)	
Acute respiratory failure	98 (15.8%)	72 (14.1%)	
Malignancy	116 (18.7%)	77 (15.1%)	
Heart disease	58 (9.3%)	42 (8.2%)	
Trauma	65 (10.5%)	65 (12.7%)	
Intoxication	30 (4.8%)	44 (8.6%)	
Others	151 (24.4%)	115 (22.5%)	

We did not find any notable differences in ICU outcomes of patients who died, specifically regarding whether patients were admitted to ICU in business or off-hours (p > 0.05). Moreover, there were no significant differences in patients who died regarding admission times, age, gender or ICU scores including GCS, SOFA or APACHE II (Table 3).

**Table 3:** Assessment of general characteristics of death cases according to admission times Acute Physiology and Chronic Health Evaluation II (APACHE II); Glasgow Coma Scale (GCS); Sequential Organ Failure Assessment (SOFA); intensive care unit (ICU); mechanic ventilation (MV); standard deviation (SD) Others refer to post-operative, infectious disease, kidney failure and gastrointestinal disease; Business hours referred to 8:00-16:59 on weekdays; Off-hours referred to 17:00-07:59 on weekdays and weekends (from 00:00 Saturday to 23:59 Sunday).

Death Cases (n=474)	Business hours (n=145)	Off-hours (n=329)	
	Mean±SD	Mean±SD	p
Age (years)	63.11 ± 18.40	63.00 ± 1769	0.953
Length of ICU stay (days)	8.56 ± 11.80	7.71 ± 8.56	0.434
Length of MV stay (days)	6.19 ± 9.78	5.95 ± 9.17	0.791
APACHE II	23,63 ± 5,12	24,06 ± 4,86	0.160
SOFA	8.25 ± 3.06	8.63 ± 3.27	0.238
GCS	7.89 ± 4.36	7.76 ± 4.23	0.766
	n (%)	n (%)	p
Gender			0.585
Male	85 (58.6%)	184 (55.9%)	
Female	60 (41.3%)	145 (44.0%)	
Admission period			0.811
Period I (10.03.2020-31.12.2020)	90 (62.0%)	208 (63.2%)	
Period II (01.01.2021-31.12.2022)	55 (38.0%)	121 (36.8%)	
Primary reason for admission			0.749
Cerebrovascular disease	32 (22.0%)	65 (19.7%)	
Acute respiratory failure	26 (17.9%)	58 (17.6%)	
Malignancy	20 (13.7%)	60 (18.2%)	
Heart disease	18 (12.4%)	31 (9.4%)	
Trauma	12 (8.2%)	37 (11.2%)	
Intoxication	2 (1.3%)	3 (0.09%)	
Others	35 (24.1%)	75 (22.7%)	

Table 4 provides a multivariate logistic regression analysis showing the association of baseline characteristics of patients with ICU mortality. Multivariate logistic regression analysis included regressors such as age, sex, SOFA, APACHE II and GCS scores, length of ICU and MV time (day), the primary reason for admission and admission time. The multivariate model exhibits statistical significance and explains/calculates 39.8% of the variability in mortality as a dependent variable (p < 0.001, r2: 40.2). The multivariate logistic regression analysis revealed that APACHE II (OR: 1.11, 95% CI: 1.06 to 1.15, p<0.01), SOFA (OR: 1.21, 95% CI: 1.10 to 1.31, p<0.01) and GCS (OR: 0.88, 95% CI: 0.84 to 0.92, p<0.01) scores were significantly associated with mortality. Additionally, the risk of mortality was 2.60 times greater for ICU admissions occurring during "off-hours" in comparison to those during "business hours" (OR: 2.63, 95% CI: 1.91-3.67, <0.01). However, no significant correlation was found whether patients were admitted to ICU during the first or second period of the COVID-19 pandemic.

**Table 4:** Multivariate logistic regression analysis showing the association of baseline characteristics of patients with intensive care unit mortality. Acute Physiology and Chronic Health Evaluation II (APACHE II); Glasgow Coma Scale (GCS); Sequential Organ Failure Assessment (SOFA); intensive care unit (ICU); mechanic ventilation (MV); standard deviation (SD) Others refer to post-operative, infectious disease, kidney failure and gastrointestinal disease; Business hours referred to 8:00-16:59 on weekdays; Off-hours referred to 17:00-07:59 on weekdays and weekends (from 00:00 Saturday to 23:59 Sunday). * Represents reference variable.

	Death (n=474)	Non-death (n=653)	OR (95% CI)	p
Age (yr)	63.1±18.2	55.5±20.9	1.01 (0.99–1.02)	0.205
Sex				
Male	269 (56.75%)	363 (55.6%)	1.22 (0.88-1.69)	0.224
Female*	205 (43.25%)	290 (44.4%)	Ref (1)	
APACHE II score	23.1±5.47	15.5±5.81	1.11 (1.06-1.15)	<0.001
GCS score	7.80±4.27	11.9±3.76	0.88 (0.84-0.92)	<0.001
SOFA score	8.51±3.21	4.75±2.67	1.21 (1.10-1.31)	<0.001
Length of MV time (day)	6.02±9.35	2.58±5.58	0.05 (1.10-1.23)	<0.001
Length of ICU time (day)	7.97±10.93	6.35±921	1.10 (1.10-1.23)	<0.001
Primary reason for admission				
Cerebrovascular disease	97 (20.5%)	97 (14.9%)	1.12 (0.67-1.88)	0.647
Acute respiratory failure	84 (17.8%)	86 (13.3%)	1.33 (0.67-1.90)	0.463
Malignancy	80 (16.9%)	113 (17.3%)	1.84 (1.11-3.06)	0.018
Heart disease	49 (10.3%)	51 (7.8%)	1.63 (0.99-3.38)	0.051
Trauma	49 (10.3%)	81 (12.4%)	0.98 (0.52-1.82)	0.956
İntoxication	5 (1%)	61 (9.4%)	0.34 (0.10-1.18)	0.088
Other*	110 (23.2%)	156 (23.9%)	Ref (1)	
Admission time				
Business hours*	145 (30.6%)	364 (55.7%)	Ref (1)	
Off-hours	329 (69.4%)	289 (44.3%)	2.63 (1.91-3.67)	<0.01
Admission period				
Period I (10.03.2020-31.12.2020)	298 (62.9%)	415 (63.6%)	0.80 (0.56-1.15)	0.248
Period II (01.01.2021-31.12.2022)*	176 (37.1%)	238 (36.4%)	Ref (1)	

## Discussion

Our findings indicate that there was no statistically significant variance in the mortality rate among non-COVID-19 patients between the first and second periods of the COVID-19 pandemic. Our study revealed that ICU admissions during "off-hours" were associated with elevated mortality rates. These observations can be attributed to variations in disease severity, as our results demonstrated that disease severity scores including APACHE II and SOFA were significantly higher in patients admitted to ICU on off-hours. However, when comparing the admission time of patients who died regarding APACHE II scores, SOFA scores, the primary reason for admission, and baseline characteristics, no significant difference was found between the off and business hours. The results of this study revealed that higher APACHE II and SOFA scores, lower GCS scores, admissions to the ICU during "off-hours", and longer MV and ICU stay time were significantly associated with mortality.

The COVID-19 pandemic is a healthcare crisis, leading to unprecedented impact on healthcare services, notable morbidity and mortality of the public and healthcare workers, economic repercussions, and significant psychological effects. To reduce the risk of viral transmission from person to person during the pandemic, governments introduced various measures such as lockdowns and restrictions, along with strategies such as “social distancing” and “self-isolation”. Healthcare professionals who are responding to a global health crisis have become targets in the fight against the pandemic which was always unexpected. Moreover, with the COVID-19 pandemic, there have been serious changes in ICU practice, as in every field of medicine. ICU staff must also continue to maintain their personal responsibilities and treat non-COVID patients while struggling with infection and death, excessive financial hardships, stress related to known and particularly unknown information, and fear of uncertainty regarding continued impact. Healthcare providers grappled with the dual task of balancing the care of COVID-19 and non-COVID patients, all the while maintaining the delivery of healthcare services characterized by high-quality standards [19]. ICU staff experienced emotional exhaustion, which may lead to medical errors, lack of empathy at times, decreased productivity, and higher turnover rates. It is reasonable to presume that the healthcare staff's physical and psychological well-being may have influenced ICU outcomes during the first period of restrictions. One can legitimately assume that the physical and psychological effects of the restrictions on healthcare staff during the first period may have impacted ICU outcomes. When we segmented the pandemic period into two distinct phases within our study, no significant difference was observed in terms of mortality rates when comparing the first period of the pandemic and the second period, characterized by the relaxation of certain restrictions and the resumption of elective surgeries. Considering the results of our study, healthcare services were not affected in non-COVID-19 ICUs.

In several research studies, it has been established that elevated mortality prediction test scores including Simplified Acute Physiology Score II are discernible among patients admitted to ICUs during off-hours, particularly from midnight until the early morning. Moreover, these studies have reported that high scores can predict ICU mortality [20,21]. When investigating the underlying factors contributing to these elevated mortality rates, it becomes evident that there exist proportional differences in terms of comorbidity and urgency levels between patients admitted to ICU during off-hours and those admitted during business hours. In a previous study investigating the effect of admission time on mortality, it was reported that admission to the ICU during nighttime was associated with a higher mortality rate, which could be explained by differences in staffing coverage [22]. In another study conducted by Sheu et al., the authors suggested that non-office-hour admissions to the ICU were not associated with poorer ICU, hospital, and ventilator outcomes, compared with office-hour admissions [23]. Another study showed that there is no significant relationship between the admission time to the emergency department and treatment outcomes. It has been reported that the admission time does not impact the length of stay in the emergency department, the duration of ICU or hospital stays, the time spent on mechanical ventilation, or the mortality rate for those admitted to the ICU [24]. The retrospective design of the studies investigating the influence of admission times to the ICU on mortality impedes the generalizability of the results and the debate continues on whether admission times to the ICU are associated with mortality rate.

In this present study, we found that patients hospitalized after standard working hours exhibited higher APACHE II and SOFA scores, which subsequently correlated with an increased mortality rate. The APACHE II and SOFA scores are widely recognized scoring systems that have been employed for many years to assess the condition and predict mortality in non-COVID-19 critically ill patients [25]. The APACHE II score categorizes the severity of illness in ICU patients by considering factors such as age, medical history, and various physiological measurements [16]. The SOFA score evaluates organ dysfunction, morbidity, and ICU-related mortality based on the functioning levels of six organ systems: respiratory, circulatory, renal, hematologic, hepatic, and central nervous systems [17]. The results of our study are consistent with the literature that patients with higher SOFA and APACHE II scores had a higher mortality rate [25,26].

A common misunderstanding about mortality in ICUs is that patients frequently die during unconventional working hours, particularly in the early morning when healthcare personnel's alertness may tend to decline. Nonetheless, a study revealed no significant variation in terms of mortality rates within ICU when analyzed across different days [27]. However, while not statistically significant, it was noted that deaths were relatively more frequent between the hours of 16:00 to 24:00. It is important to consider that patients' involvement in decisions related to their own end-of-life choices and the timing of such decisions may be influenced by cultural variations and disparities in beliefs about death among different countries [28]. These factors could potentially impact the results observed in the study.

In a previous study, Jules Mesnier et al. reported a reduction in hospital admissions after the implementation of lockdown measures during the COVID-19 pandemic [29]. Notably, this decrease was consistent across patient characteristics and the regional prevalence of COVID-19. These findings hold considerable significance for healthcare practitioners and policymakers seeking to enhance the robustness of healthcare systems and hospitals in the face of pandemics or analogous acute disruptions, including extreme climatic events. The reduction in hospital admissions after the implementation of lockdown measures during COVID-19 can be attributed to various factors, though their exact nature remains speculative. Similar outcomes have been reported in other studies, yet the underlying causes remain uncertain and sometimes contradictory [30].

This study had some limitations. Firstly, the study design was retrospective. Since retrospective studies rely on the review of medical records that were not initially structured for research purposes, it is expected that certain data may be absent or of suboptimal quality. We encountered a deficiency in fundamental demographic information, including ethnicity, smoking habits, and co-morbidities. However, it is difficult to plan a prospective study to estimate the mortality rate during the pandemic with a larger sample size. Secondly, admission time to the ICU was divided into off-hours and business hours. The timing of admission to the ICU could be also designed as weekdays and weekends. Third, situations such as curfews and restrictions, strict rules regarding social distancing and protection measures may also have affected the period it takes for patients to be treated and be transferred to the hospital during the pandemic period. Moreover, these restriction periods were different regarding the countries. Additionally, we investigated cumulative mortality and examined factors such as admission and time of death in relation to the intensity of the pandemic, rather than focusing solely on the time elapsed since admission. Another limitation is that that some patients died after discharge from the ICU, and we have not been able to follow up on these cases, which could affect the results of this study. The workforce during office hours and out-of-office hours could differ according to the staffing and financial resources in different countries. Lastly, although COVID-19 patients were not included in the study, the streamlining workflows for swift diagnosis, isolation, clinical management, and infection control is not only pertinent to COVID-19 patients but also to non-COVID-19 patients. To address these challenges, we must gain a deeper understanding of the true limitations and vulnerabilities within our healthcare systems. To unravel this intricate web of interconnected causal elements, it is advisable to extend the study timeframe to include the months following the conclusion of the lockdown.

## Conclusions

In conclusion, our study found that the mortality rate was higher in non-COVID-19 patients admitted to the ICU during off-hours. Additionally, higher APACHE II and SOFA scores, lower GCS scores, and longer MV and ICU stay times were significantly associated with mortality in the ICU. However, no significant difference was found in the mortality rate between the admissions during the first and second periods of the COVID-19 pandemic. Ultimately, broadening the scope of our analysis to include admissions for other medical conditions would provide further valuable insights into the contrasting influences of generic and disease-specific factors, the nuances surrounding admission times, and the impact of local healthcare organizations, as well as the significance of clinical features upon admission.
